# The higher mortality associated with low serum albumin is dependent on systemic inflammation in end-stage kidney disease

**DOI:** 10.1371/journal.pone.0190410

**Published:** 2018-01-03

**Authors:** Filipa Caeiro Alves, Jia Sun, Abdul Rashid Qureshi, Lu Dai, Sunna Snaedal, Peter Bárány, Olof Heimbürger, Bengt Lindholm, Peter Stenvinkel

**Affiliations:** 1 Hospital Espírito Santo, Évora, Portugal; 2 Renal Medicine and Baxter Novum, CLINTEC, Karolinska Institutet, Stockholm, Sweden; 3 Landspitali University Hospital, Reykjavik, Iceland; The Pennsylvania State University, UNITED STATES

## Abstract

**Background:**

The correlation of low serum albumin with mortality in patients with chronic kidney disease (CKD) is partly linked to its association with systemic inflammation. However, it is not clear to what extent albumin's correlation with mortality depends on concomitant systemic inflammation. Here we addressed this question in patients with CKD stage 5.

**Methods:**

Serum albumin (S-Alb), systemic inflammation (high-sensitive C-reactive protein, hsCRP), cardiovascular disease (CVD) and nutritional status (subjective global assessment, SGA) were assessed at baseline in 822 patients: 523 incident dialysis patients, 212 prevalent hemodialysis (HD) and 87 prevalent peritoneal dialysis (PD) patients. Patients were divided into four groups according to hsCRP and S-Alb in each cohort: Group 1 –normal S-Alb and normal hsCRP (reference); Group 2 –low S-Alb and normal hsCRP; Group 3—normal S-Alb and high hsCRP; Group 4—low S-Alb and high hsCRP. Survival over 60 months was analyzed.

**Results:**

In Cox analysis, Group 4 had an increased mortality risk (adjusted Hazard ratio (95% confidence interval): 1.62 (1.06–2.47); p = 0.02) whereas the augmented mortality risks for Groups 2 and 3 in univariate analyses were not significant after adjustments for age, gender, blood pressure, diabetes mellitus, smoking, SGA, renal function and renal replacement technique.

**Conclusions:**

Whereas mortality risk was increased in CKD stage 5 patients with low S-Alb and high CRP, it was not increased in patients with low S-Alb and normal CRP. Our observation suggests that inflammatory status should be taken into account when using S-albumin for risk assessment in CKD stage 5 patients.

## Introduction

Hypoalbuminemia is a frequent feature of chronic kidney disease (CKD) [1, 2] and associates with poor clinical outcome [3, 4]. Serum albumin (S-Alb) was former considered a biomarker of visceral protein and immunocompetence status, fundamental to nutritional assessment [5]. However, recent studies show that low S-Alb rather reflects a state of persistent inflammation [6–8] and has limited value as a marker of nutritional status [9, 10]. Indeed, in the Minnesota study [11], an experiment of human semi-starvation in healthy individuals, S-Alb did not reflect nutritional changes nor body weight variation. A similar scenario was reported in patients with anorexia nervosa [12]. Despite the multiple essential functions of albumin (maintaining colloid osmotic pressure, transporting nutrients, hormones and drugs, serving as a free radical scavenger and having antithrombotic properties [13]) patients with the rare congenital disorder analbuminemia have mild or even absent symptoms [14].

Persistent inflammation, usually assessed by C-reactive protein (CRP) levels, is a characteristic feature in CKD, caused by multiple factors of the toxic uremic milieu and the dialysis technique itself [13, 15–17]. Inflammation contributes to decreased S-Alb levels [18] and plays a central role in the Malnutrition, Inflammation and Atherosclerosis syndrome (MIA) [15, 19], which associates with poor cardiovascular outcomes [20]. There is no consensus regarding the appropriate cut-off concentration for CRP in CKD, making it problematic to ascertain and understand the magnitude, variability and consequences of uremic inflammation [16, 21, 22].

Based on the inflammation-catalyst hypothesis [23]; i.e., persistent inflammation exacerbates the effect of other risk factors in the uremic milieu, we hypothesized that inflammation would have a catalytic effect on hypoalbuminemia as a risk predictor. Thus, the effect of sustained inflammation on S-Alb levels, through inhibition of its synthesis and induction of its catabolism [24], would be mainly responsible for the documented association between mortality and hypoalbuminemia [7, 13]. Albumin has a longer half-life and lower variability than traditional inflammatory markers, such as CRP [22], and may better reflect sustained inflammation. Therefore, the operational hypothesis of the current study is that S-Alb and CRP–two biochemical parameters easily accessible in clinical daily practice–should be used together for an improved risk assessment. We tested this hypothesis in incident and prevalent dialysis patients.

## Materials and methods

### Patients and study design

This prospective observational study was conducted in 822 patients, comprising data from four independent cohorts described in detail previously [25–28]. Incident dialysis patients were recruited among consecutive patients initiating dialysis at the Department of Renal Medicine, Karolinska University Hospital, from June 1994 until November, 2014 as part of the ongoing MIA (Malnutrition, Inflammation Atherosclerosis) study; see [15, 19, 26] for further details regarding all aspects of study design. 51 prevalent PD patients were recruited from May 2008 to January 2011 in the MIMICK 2 (Mapping of Inflammation Markers in Chronic Kidney Disease 2) study among all prevalent PD patients treated in Stockholm; see [27] for further details regarding all aspects of study design. 174 prevalent HD patients were recruited from November 2003 until September 2004 in the MIMICK 1 (Mapping of Inflammation Markers in Chronic Kidney Disease 1) study among all prevalent HD patients treated in Stockholm; see [22] for further details regarding all aspects of study design. 36 prevalent PD patients and 38 prevalent HD patients were recruited from May 2009 to June, 2016 in an ongoing study of vascular changes in ESRD patients undergoing living donor renal transplantation (LD-Rtx study); see [28] for further details. They were there upon redistributed according to the renal replacement status in 523 incident dialysis patients, 212 prevalent hemodialysis (HD) patients and 87 prevalent peritoneal dialysis (PD) patients. Patients´ characteristics at baseline in these three cohorts are displayed in **S1 Table**.

The patients were divided in normal or low S-Alb, using the cutoff of 35 g/L, and in normal or high high-sensitive CRP (hsCRP), using the cutoff of 3 mg/L, and consequently divided into four groups: Group 1 (n = 200)–normal S-Alb and normal hsCRP; Group 2 (n = 160)—low S-Alb and normal hsCRP; Group 3 (n = 172)–normal S-Alb and high hsCRP; Group 4 (n = 290)—low S-Alb and high hsCRP. Typical cardiovascular risk factors (i.e., age, gender, hypertension, diabetes mellitus, dyslipidemia, smoking) and atypical, or CKD-related cardiovascular risk factors, i.e., anemia, bone-mineral disorder, inflammation, protein-energy wasting (PEW; subjective global assessment) and oxidative stress, where compared among the four groups (**Table 1**). Survival was determined after a follow-up period of 60 months. There were no patients lost to follow-up. The Ethics Committee of the Karolinska Institute, Stockholm, Sweden, approved the study protocol. Informed written consent was obtained from each patient.

**Table 1:** Demographic, clinical and laboratory characteristics of 822 CKD stage 5 patients divided into four groups defined according to levels of serum albumin and hsCRP.

Characteristics	Group 1 Normal albumin Normal hsCRP (n = 200)	Group 2 Low albumin Normal hsCRP (n = 160)	Group 3 Normal albumin High hsCRP (n = 172)	Group 4 Low albumin High hsCRP (n = 290)	p value
**CKD5/HD/PD** (%)	65/24/11	63/19/19	62/34/5	64/26/9	0.001
**Residual renal function** (ml/min/1.73 m^2^)	5.3 (0.0–9.4)	4.9 (0.0–10.1)	5.0 (0.0–9.5)	5.2 (0.0–8.9)	0.92
**Urinary albumin excretion** (mg/24h) ^a^	16023 (145–4008)	2228 (225–7945)	1262 (86–4443)	2380 (349–6220)	**<0.001**
**Traditional cardiovascular risk factors**					
**Age** (years)	47 (26–70)	55 (32–74)	59 (37–76)	61 (39–75)	**<0.001**
**Gender** (male %)	61	50	67	64	**0.01**
**Cardiovascular disease** (%)	24	29	48	48	**<0.001**
**Diabetes mellitus** (%)	12	33	24	34	**<0.001**
**Smoking** (%) ^b^	39	43	60	62	**<0.001**
**Systolic BP** (mmHg) ^c^	140 (120–172)	147 (120–180)	143 (112–171)	145 (110–180)	0.07
**Diastolic BP** (mmHg) ^c^	85 (70–101)	87 (70–103)	82 (65–97)	84 (65–103)	0.14
**BMI** (kg/m^2^)	23.5 (19.1–29.0)	24.6 (20.3–30.2)	24.7 (20.4–30.6)	24.0 (18.9–30.5)	**0.003**
**Cholesterol** (mmol/L) ^d^	4.6 (3.3–6.5)	4.8 (3.3–6.9)	4.6 (3.4–6.4)	4.6 (3.0–7.0)	0.63
**Triglycerides** (mmol/L) ^e^	1.5 (0.8–2.8)	1.5 (0.7–2.8)	1.7 (0.8–3.5)	1.7 (0.9–3.2)	**0.004**
**Uremia related cardiovascular risk factors**					
**Hemoglobin** (g/L)	114 (95–131)	109 (93–129)	110 (92–128)	106 (86–127)	**<0.001**
**Ferritin** (ng/ml) ^f^	288 (93–753)	269 (58–561)	335 (99–798)	330 (104–897)	**0.007**
**Serum albumin** (g/L)	38 (35–42)	32 (26–33)	37 (35–41)	30 (24–34)	**<0.001**
**SGA score >1** (%)	27	28	37	48	**<0.001**
**Handgrip strength** (% of control)	92 (56–121)	83 (58–122)	77 (48–114)	67 (41–103)	**<0.001**
**PTH** (ng/L) ^g^	251 (48–631)	220 (70–515)	207 (56–638)	248 (56–610)	0.26
**Calcium** (mmol/L) ^h^	2.42 (2.13–2.71)	2.31 (2.05–2.69)	2.51 (2.18–2.75)	2.42 (2.05–2.75)	**<0.001**
**Phosphate (**mmol/L) ^i^	1.8 (1.2–2.6)	1.8 (1.2–2.5)	1.8 (1.3–2.6)	1.9 (1.2–2.6)	0.48
**hsCRP** (mg/L)	1.1 (0.3–2.5)	1.2 (0.2–2.6)	7.9 (3.8–28.0)	14.0 (4.2–58.3)	**<0.001**
**IL-6** (pg/ml) ^j^	2.8 (0.5–8.4)	3.8 (0.9–9.9)	6.7 (2.1–15.8)	9.6 (4.2–25.4)	**<0.001**
**TNF** (pg/ml) ^k^	11.6 (7.5–18.2)	10.9 (6.6–18.0)	12.9 (7.8–19.4)	14.4 (8.5–24.7)	**<0.001**
**VCAM-1** (ng/ml) ^l^	1205 (810–1895)	1301 (873–1700)	1398 (824–2027)	1574 (1043–2795)	**<0.001**
**8-OHdG** (ng/ml) ^m^	0.63 (0.15–1.56)	0.54 (0.14–1.39)	0.73 (0.31–1.72)	0.85 (0.42–1.88)	**<0.001**

**Abbreviations:** BP, blood pressure; BMI, body mass index; SGA, subjective global assessment of nutritional status; PTH, parathyroid hormone; hsCRP, high-sensitivity C-reactive protein; IL-6, interleukin-6; TNF, tumor necrosis factor; VCAM-1, vascular cellular adhesion molecule-1; 8-OHdG, 8-hydroxy-2'-deoxyguanosine. **Low albumin** was defined as S-Alb levels <35g/L, **High hsCRP** was defined as hsCRP levels ≥3mg/L, **Normal albumin** was defined as S-Alb levels ≥35g/L and **Normal hsCRP** levels was defined as hsCRP levels <3 mg/L. Values presented as median (range 10^th^ to 90^th^ percentiles).

^a^ n = 300

^b^ n = 678

^c^ n = 703

^d^ n = 817

^e^ n = 815

^f^ n = 687

^g^ n = 725

^h^ n = 790

^i^ n = 785

^j^ n = 789

^k^ n = 754

^l^ n = 480

^m^ n = 528.

### Clinical, anthropometric and nutritional evaluation

Cardiovascular disease (CVD) was defined by medical history or clinical findings of cardiac, cerebrovascular or peripheral arterial disease. Smoking habits were recorded as current and former smokers and non-smokers. Anthropometric measurements were obtained at the baseline, after the dialysis session in case of prevalent HD patients. Body mass index (BMI) was calculated. Handgrip strength (HGS) was measured in both hands using a Harpenden Handgrip Dynamometer (Yamar, Jackson, MI, USA). The dominant hand measurement was used in our analysis due to the regular presence of arteriovenous fistulas in the non-dominant arm. Values for HGS were expressed as percentage of healthy subjects, adjusted for gender.

Nutritional status was evaluated using the 6 components of the subjective global assessment (SGA) questionnaire: patient’s history of weight loss, anorexia and vomiting, and physician’s grading of muscle wasting, presence of oedema and loss of subcutaneous fat [29]. Poor nutritional status was defined as a SGA>1. Arterial systolic and diastolic blood pressure was measured three times in the morning after a 15-minute resting period.

### Biochemical analysis

Venous blood samples were collected at baseline, before the dialysis session in hemodialysis patients. The plasma was separated and samples were kept frozen at –70° C if not analysed immediately. S-Alb concentration was measured with bromocresol purple method; hsCRP concentration by nephelometry; and concentrations of hemoglobin, creatinine, ferritin, cholesterol, triglycerides, parathyroid (PTH), calcium and phosphate were determined by routine methods at the Department of Laboratory Medicine, Karolinska University Hospital. Interleukin-6 (IL-6) and plasma tumor necrosis factor (TNF) were analyzed by enzyme-labeled chemiluminescent assay (Immulite, DPC, Los Angeles, CA); serum 8-OHdG by commercial competitive enzyme-linked immunosorbent assay (ELISA) kit (Japan Institute for the Control of Aging, Shizuoka, Japan); and vascular cellular adhesion molecule 1 (VCAM-1) by commercial ELISA kits.

### Statistical analysis

Continuous variables were expressed as median with range 10^th^ to 90^th^ percentile and Chi-square was used for nominal variables. Nonparametric ANOVA were used for three or more groups: Kruskal-Wallis test for continuous variables, followed by Dunn’s test. Spearman rank test was used for correlation between continuous and ordinal variables. Statistical significance was set at the level of p<0.05. Imputation of missing values was performed using the function MI. The datasets were limited to ten imputations to avoid overestimation due to Monte Carlo variability. Survival analysis was performed using Kaplan-Meier survival curves and Cox proportional hazard models. Hazard ratios with 95% confidence interval for mortality were adjusted for age, gender, CVD, diabetes mellitus, smoking, residual renal function and treatment modality. Cox model assumptions were tested using Schoenfeld residuals plot for proportional hazards. Statistical analyses were performed using statistical software SAS version 9.4 (SAS Campus Drive, Cary, NC, USA) and Stata 15.0 (Stata Corporation, College Station, TX, USA).

## Results

Demographics and phenotype of the patients in the four groups are shown in **Table 1**. As expected, Group 4 patients (low S-Alb and high hsCRP) were older and had a higher prevalence of CVD (48%). The prevalence of diabetes mellitus was higher in Groups 2 (33%) and 4 (34%); i.e. the low S-Alb groups. Traditional cardiovascular risk factors systolic arterial blood pressure, and total cholesterol did not differ significantly between Group 4 and Group 1. Urinary albumin excretion (only available in incident dialysis and prevalent PD patients) was higher in the two groups with lowest levels of S-Alb (Groups 2 and 4) but did not differ significantly between the two groups. The lowest haemoglobin levels were found in Group 4 and the higher ferritin levels in both groups with higher hsCRP (Groups 3 and 4). No significant differences in PTH and phosphate levels were observed among the groups. The inflammatory markers hsCRP, IL-6, TNF and VCAM-1 and the oxidative stress marker 8-OHdG were significantly higher in Groups 3 and 4.

A correlation matrix is presented in **Table 2**. S-Alb and hsCRP correlated significantly with most biomarkers and clinical parameters but not with residual renal function, PTH, phosphate or cholesterol.

**Table 2:** Univariate Spearman’s Rho correlations of S-albumin and hsCRP with other parameters in 822 patients with CKD stage 5.

Characteristics ^d^	S-Albumin	hsCRP
**Age**	**- 0.17**^**c**^	**0.30**^**c**^
**Gender**	0.04	**0.07**^**a**^
**Residual renal function**	- 0.06	- 0.02
**CVD**	**- 0.11**^**c**^	**0.29**^**c**^
**Diabetes mellitus**	**- 0.25**^**c**^	**0.09**^**b**^
**Smoking**	**- 0.08**^**a**^	**0.23**^**c**^
**Systolic BP**	**- 0.10**^**b**^	-0.04
**Diastolic BP**	-0.06	**-0.09**^**a**^
**MAP**	**- 0.09**^**a**^	- 0.05
**Pulse Pressure**	**- 0.12**^**b**^	0.04
**BMI**	0.01	**0.07**^**a**^
**SGA score >1**	**- 0.16**^**c**^	**0.23**^**c**^
**%Handgrip strength**	**0.23**^**c**^	**- 0.32**^**c**^
**Hemoglobin**	**0.20**^**c**^	**- 0.15**^**c**^
**Ferritin**	0.00	**0.16**^**c**^
**PTH**	- 0.01	0.00
**Calcium**	**0.18**^**c**^	**0.15**^**a**^
**Phosphate**	- 0.03	0.01
**Cholesterol**	- 0.01	- 0.04
**Triglycerides**	0.00	**0.14**^**c**^
**IL-6**	**- 0.38**^**c**^	**0.64**^**c**^
**TNF**	**- 0.14**^**c**^	**0.25**^**c**^
**VCAM-1**	**- 0.24**^**c**^	**0.30**^**c**^
**8-OHdG**	- 0.07	**0.36**^**c**^

**Abbreviations:** BP, blood pressure; hsCRP, high-sensitivity C-reactive protein); RRF, residual renal function; CVD, cardiovascular disease; MAP, mean arterial pressure; BMI, body mass index; SGA, subjective global assessment of nutritional status: PTH, parathyroid hormone; IL-6; interleukin-6; TNF, tumor necrosis factor; VCAM-1, vascular cellular adhesion molecule-1; 8-OHdG, 8-hydroxy-2-deoxyguanosine.

^**a**^ p<0.05

^**b**^ p<0.01

^**c**^ p<0.001

^d^ for number of patients for each parameter see Table 1

### Anthropometric and nutritional evaluation

**Fig 1:**
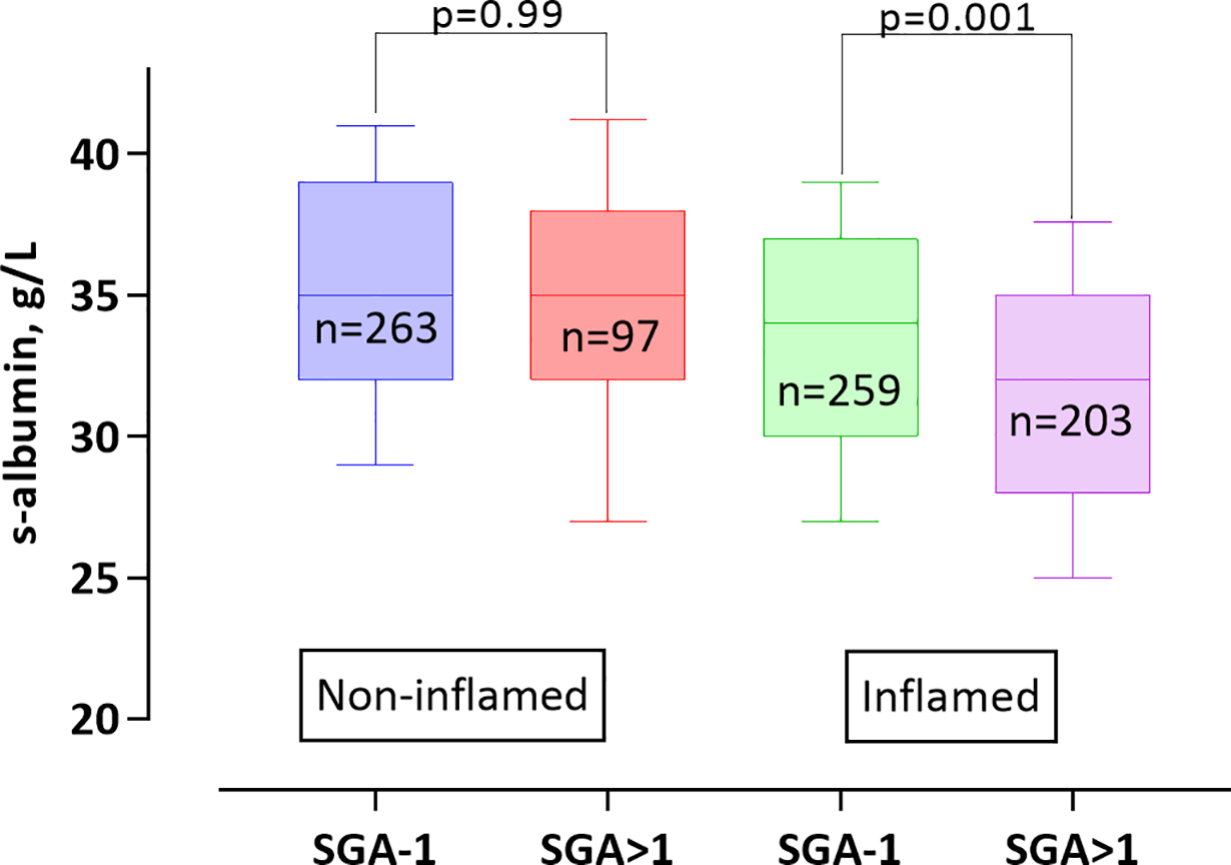
Association between S- albumin and subjective global assessment score (SGA) in 822 CKD stage 5 patients, according to inflammatory status. Inflamed and non-inflamed were defined according to hsCRP concentrations above or below 3 mg/L.

BMI significantly differed between the four groups with the lowest values found in Group 1. Group 4 had significantly lower HGS than Group 1, 2 and 3 (67% vs 92%, 83% and 77% respectively; p<0.001) and the prevalence of PEW (SGA>1) was significantly higher in Group 4 than in Groups 1, 2 and 3 (48% vs. 27%, 28% and 37%, respectively; p<0.001). When assessing S-Alb in relation to nutritional status in inflamed (Groups 3–4) and non-inflamed (Groups 1–2) patients, the presence of PEW (SGA>1) was associated with presence of a low S-Alb level only among the inflamed patients **(Fig 1).** In contrast, in the non-inflamed patients, presence of a low S-Alb level did not differ between patients with PEW (SGA>1) and well-nourished patients.

### Survival analysis

**Fig 2:**
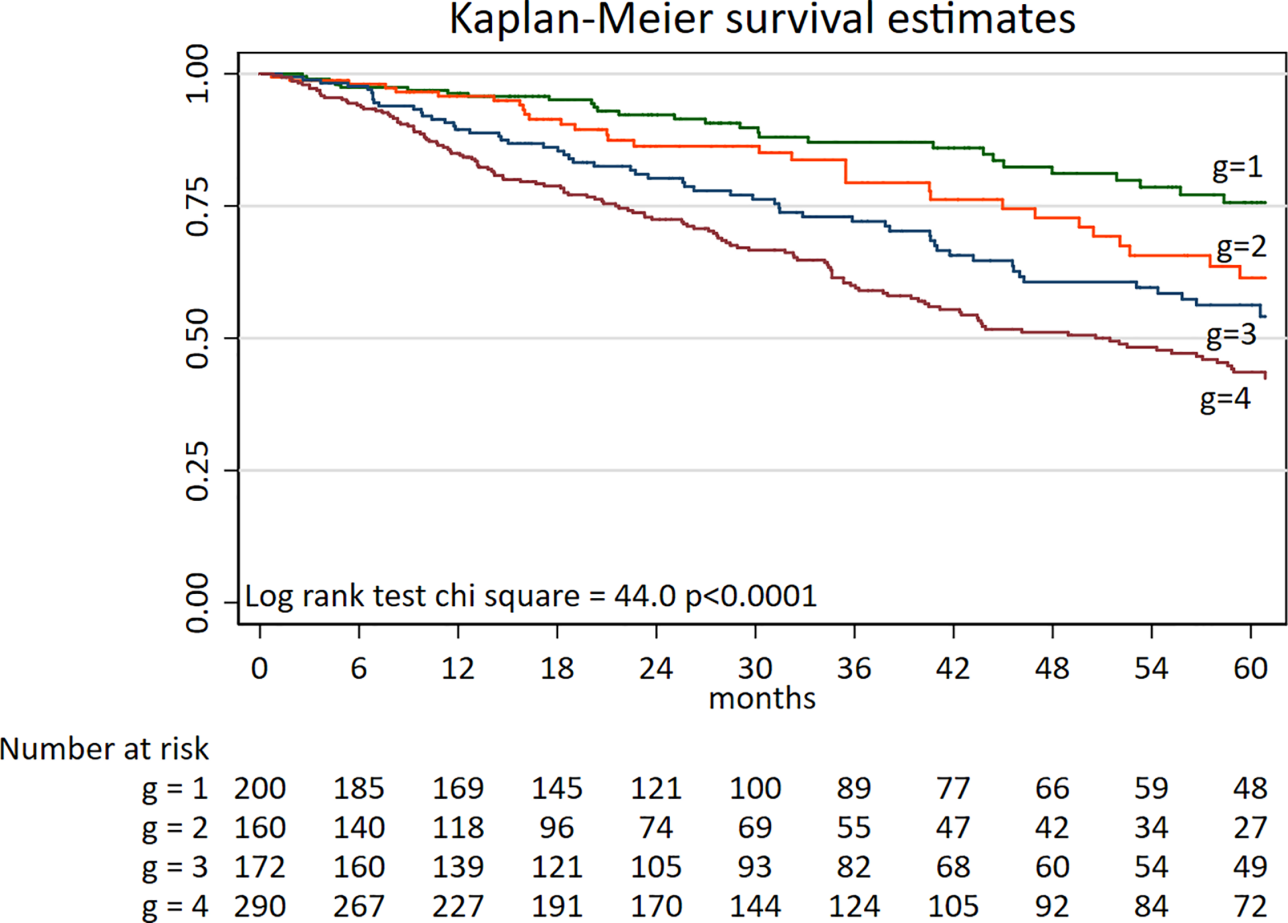
Kaplan-Meier survival curves during 60 months follow-up in 822 CKD stage 5 patients divided into four groups according to levels of hsCRP (high ≥3mg/L, or normal <3mg/L) and S-Alb (low <35 g/L, or normal ≥35g/L). Group 1 (n = 200): Normal S-Alb and normal hsCRP; Group 2 (n = 160): Low S-Alb and normal hsCRP; Group 3 (n = 172): Normal S-Alb and high hsCRP; Group 4 (n = 290) Low S-Alb and high hsCRP.

Patients' mortality (by Kaplan-Meier) **(Fig 2)** was significantly higher in Group 4 (p<0.001). In contrast, no significant difference in mortality was observed for Groups 2 and 3 compared with the reference group (Group 1). In Cox Proportional Hazard analysis adjusted for age, gender, CVD, diabetes mellitus, smoking, residual renal function and treatment modality, Group 4 demonstrated higher mortality risk compared to the reference group (HR 1.62 (1.06–2.47); p = 0.02), while Group 2 and 3 did not associate with increased mortality risk as compared to Group 1 **(Table 3**).

**Table 3:** All-cause mortality risk associated with low S-Alb <35 g/L and high hsCRP ≥3 mg/L (Group 4) during 60 months of follow-up.

	Crude HR (95% CI)	p value	Adjusted imputed HR (95% CI)	p value
**Group 2** Low albumin Normal hsCRP	**1.63 (0.98–2.73)**	**0.05**	1.04 (0.61–1.76)	0.89
**Group 3** Normal albumin High hsCRP	**2.30 (1.46–3.62)**	**<0.001**	1.27 (0.80–2.03)	0.29
**Group 4** Low albumin High hsCRP	**3.35 (2.22–5.04)**	**<0.001**	**1.62 (1.06–2.47)**	**0.02**

Data are presented as hazard ratios (HR) with 95% confidence interval (CI) crude and adjusted for confounding factors (age, gender, CVD, DM, smoking, SGA, GFR and renal replacement technique), using Group 1 as reference. Imputed smoking status was used in 144 patients.

These results remained in principle unchanged following several additional analyses serving as sensitivity tests (**S2 Table, S3 Table, S4 Table, S5 Table, S6 Table**). Thus, association of S-Alb with all-cause mortality risk remained when using median values for S-Alb and hsCRP; excluding all PD patients; analyzing S-Alb as a continuous variable for low and high CRP respectively; analyzing HR for S-Alb in the whole population without adjusting for CRP and after adjusting for CRP; adjusting for blood pressure (instead of CVD) and adjusting for %HGS. S-Alb remained associated with HR in the whole population without adjusting for CRP and after adjusting for CRP. However, when adjusting for additional confounders, including traditional CVD risk factors and uremia risk factors (age, gender, DM, BP, SGA, GFR, renal replacement modality, %HGS, Ca x PO_4_, serum cholesterol, triglycerides and hemoglobin), Group 4 (Low S-Alb and high hsCRP) was not associated with increased risk (HR 1.43 [0.94–2.20]; p = 0.11).

## Discussion

We show, for the first time, that inflammation has a catalytic effect on the genuine risk biomarker S-Alb. Thus, low S-Alb was found to be an independent risk predictor for poor outcome only in the presence of inflammation whereas hypoalbuminemia without inflammation carried a minor mortality risk. These results withstood extensive sensitivity analyses (see Supplementary material). This observation accords with recent studies demonstrating that hypoalbuminemia in the uremic milieu rather reflects persistent inflammation [6–9] than malnutrition (i.e. PEW) and that the role of albumin as a prognostic marker [3, 30] is lost after adjustments for inflammatory markers [31–33].

The concentration of S-Alb depends on many factors, such as the rate of hepatic synthesis and secretion, exchanges between intra- and extravascular compartments, lymphatic uptake, alterations of the volume of distribution and protein degradation rate, and body losses [6, 24]. During systemic inflammation, opposing the reduced albumin degradation that accompanies low intake or body losses, the catabolism of albumin is increased, leading to declining S-Alb concentrations [34]. Due to its long half-life, a single measurement of S-Alb is likely to reflect a more prolonged period or a more relevant state of uremic inflammation than the “moving targets” of CRP and IL-6 in the highly variable uremic milieu [16, 22]. In fact, we show that the two groups of inflamed subjects present different levels of inflammation and the group with higher levels of inflammatory markers is the one with low albumin. Hypoalbuminemia can also reflect malnutrition severe enough to disturb the albumin synthesis capacity needed to maintain albumin homeostasis. Because of the abovementioned central role of S-Alb in body homeostasis, many factors linked to clinical outcome are causually associated with the concentration of S-Alb, and residual confounding is therefore likely a potential limitation in the current as well as previous studies.

Even though all inflammatory markers were higher in Group 4, appropriate cut-offs of these markers for risk assessment have not been established and they are therefore not useful to stratify the degree of severity of inflammation in CKD. The increased risk in Group 3, despite being non-significant, supports that inflammation carries risk, but there is no consensus for stratification according to inflammation level. We also used median values of hsCRP (and S-Alb) in the statistical analyses, and the results remained essentially the same. Cut-offs derived from ROC curve analysis of biomarker concentrations in relation to all-cause mortality may yield meaningful cut-offs. However, the shape of the ROC curve for S-Alb was relatively flat (**S1 Fig**). Thus, the definition of an optimal cut-off value for S-Alb is uncertain. Repeated measurements of inflammatory markers, such as hsCRP, have shown to be better predictors of mortality [16] but this approach is seldom possible in clinical practice. Therefore, the combination with an established risk biomarker with a stable temporal profile, such as S-Alb, is of value in the clinical situation.

The high prevalence of CVD and PEW in the two inflamed patient groups supports the documented strong association between inflammation, atherosclerosis and malnutrition (MIA) [19, 35–37]. It is notable that despite the high overall prevalence of CVD in these patients, the traditional cardiovascular risk factors were not consistently augmented. This observation accords with the reported lack of validity of Framingham risk assessment for risk stratification in CKD [38] and the need for developing new risk scores validated for these patients [39].

It is notable that the nutritional status (according to SGA and HGS) in the group of patients with low S-Alb in an uninflamed milieu (Group 2) did not differ from the patients with normal S-Alb levels and uninflamed milieu (Group 1). The lower BMI in the group with normal S-Alb and low hsCRP, the group with better survival, are not congruent with the reported paradox of obesity being a survival factor in ESRD patients [40]. However, only 11.1% of the 822 patients were considered obese according to BMI, a lower prevalence than in most other populations [41]. Moreover, it has been demonstrated that inflammation modifies also the association between BMI and mortality [42]. A major finding of the present study is that PEW (by SGA) modifies the relationship between S-Alb/hsCRP grouping and outcome. Importantly, we observe that low S-Alb in an uninflamed milieu (Group 2) or normal S-Alb in an inflamed milieu (Group 3) predicts increased risk only in CKD5 patients with signs of PEW. Thus, like inflammation, the presence of PEW changes the risk factor profile.

**Fig 3:**
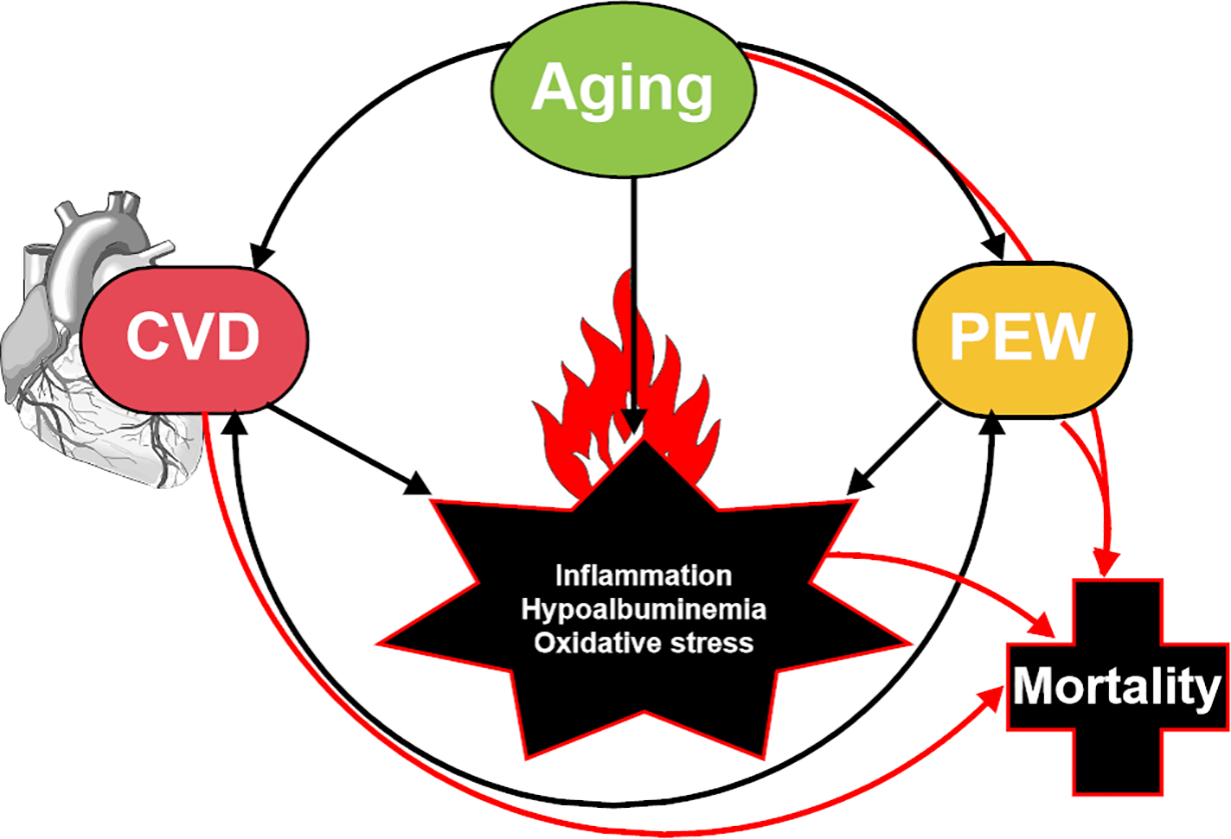
Inflammation/oxidative stress, resulting in hypoalbuminemia, interacts with cardiovascular disease (CVD) and protein-energy wasting (PEW) and together these changes contribute to the increased mortality in end-stage kidney disease.

Oxidative stress is a common feature of the inflamed uremic milieu that acts as a unifying mechanism for many cardiovascular risk factors and cardiovascular mortality [43, 44]. It promotes accelerated vascular aging via endothelial dysfunction [37], increased oxidative modifications of proteins [45] and vascular calcification [46]. Indeed, in Group 4 we report higher levels of the oxidative stress marker 8-OHdG, a major product of DNA oxidative damage that predicts increased mortality in dialysis patients [27]. Together with the increased exposure to inflammatory stimulus, CKD patients are subjected to increased susceptibility to oxidative stress caused in part by lower levels of antioxidants [43, 44]. It should be noted that S-Alb itself is a major antioxidant [13, 47], and a free-radical scavenger due to its thiol groups that act by preventing oxidative injury to both lipoproteins and the vascular wall [48]. This accords with Kim et al. [49] who showed that endothelial function markers were correlated with S-Alb. Since CRP did not normalize with albumin infusion, this suggests that the relationship between low S-Alb levels and endothelial dysfunction is intermediated by inflammation. Thus, oxidative stress (as a consequence of inflammation) combined with hypoalbuminemia [43] will lead to a vicious cycle of more deleterious consequences including deterioration of the nutritional status [50, 51]. Inflammatory cytokines promote PEW by acting directly on the gastrointestinal system or indirectly through affecting appetite and resting energy expenditure [7, 52]. Moreover, low S-Alb and congestive heart failure may promote intestinal edema [53] and disturb the absorption of essential nutrients and anti-oxidative vitamins and oligoelements [54]. These multiple interactions [55] contribute to the higher cardiovascular burden and mortality in CKD5 patients with persistent inflammation and lower albumin **(Fig 3).**

Some strengths and limitations should be considered when the results of the present study are interpreted. The study benefits from the careful phenotyping and inclusion of several traditional and non-traditional risk factors in a relatively large population of CKD5 patients. To the best of our knowledge, this is the first study in which the risk factor modifying effect of CRP on the well-established risk biomarker S-Alb is observed. The absence of patients lost to follow-up also strengthens our study. Limitations to be acknowledged are, at first, the age of patients in the four different groups is statistically different which might explain some of the differences found in anthropometric parameters. The absence of residual renal function in prevalent HD patients also limits the interpretation of its contribution to the outcomes. Our study comprised both incident and prevalent dialysis patients with somewhat different inclusion criteria. However, in the multivariate analysis, patient cohorts were taken into account, and all anthropometric and laboratory analyses were made with the same methods and techniques. Nevertheless, a number of factors influence the levels of both S-Alb and hsCRP and residual confounding remains as a potential limitation. We also acknowledge that since mortality tended to be higher in Group 3 (**Fig 2**) we cannot exclude a type-2 statistical error due to lack of power. Thus power is likely playing a role in the lack of significance in Group 3. Since inflammation markers are “moving targets”, the lack of repeated sampling of inflammatory markers limits the interpretation of the study. We recently showed that short-term variability of IL-6 and CRP strongly associated with PEW [56]. We also did not measure oxidized albumin levels, which could have had an impact on our results, as it is shown to be related with augmented oxidative stress and metabolic syndrome [57]. The quantification of albumin in peritoneal dialysis dialysate was not performed, which could be an important confounder to the albumin levels in this group of patients; however, results were essentially unchanged when we excluded PD patients. Finally, we stratified patients according to S-Alb and CRP cut-offs currently used in clinical practice; however, the best cut-off levels for risk prediction in end stage CKD patients remain to be determined. For this purpose, future studies comprising larger cohorts of patients are warranted and should take into account that the relationship between CRP and albumin concentration is non-linear.

In conclusion, a low S-Alb concentration was an independent risk factor for poor outcome in CKD stage 5 only in the presence of systemic inflammation. Based on this observation we suggest that when using S-Alb to predict mortality risk in CKD stage 5 patients, CRP should always be assessed and considered.

## Supporting information

S1 Table Baseline demographic and biochemical characteristics of 822 patients in three cohorts of incident dialysis patients, prevalent HD patients and prevalent PD patients without and with imputed data for smoking status, mean BP and %HGS. (PDF)

S2 Table All-cause mortality risk associated with low S-Alb <35 g/L and high hsCRP> 3 mg/L (Group 4) during 60 months of follow-up in CKD stage 5, without PD patients (n = 735) and without and with imputed data for smoking status, mean BP and %HGS. (PDF)

S3 Table All-cause mortality risk associated with low S-Alb <35 g/L and high hsCRP> 3 mg/L (Group 4) during 60 months of follow-up, adjusting for blood pressure instead of CVD (n = 822). (PDF)

S4 Table All-cause mortality risk associated with low S-Alb <35 g/L and high hsCRP ≥3 mg/L (Group 4) during 60 months of follow-up (n = 822) without and with adjustments for calcium x phosphate product, cholesterol, triglycerides and hemoglobin. (PDF)

S5 Table Association of continuous serum albumin with mortality risk for high and low hsCRP (n = 822). (PDF)

S6 Table All-cause mortality risk associated with serum albumin with and without adjustment for hsCRP (n = 822). (DOCX)

S1 Fig ROC analysis of serum albumin and hsCRP in relation to all-cause mortality. (PDF)

S1 Data Microsoft Excel database file of baseline, demographic and biochemical characteristics of 822 patients. (TXT)
