# Supplementary material for: The higher mortality associated with low serum albumin is dependent on systemic inflammation in end-stage kidney disease
Source: PLoS One. 2018 Jan 3;13(1):e0190410. doi: 10.1371/journal.pone.0190410 (PMC5752034; doi:10.1371/journal.pone.0190410)
Supplement: S1 Table — (PDF) [file pone.0190410.s001.pdf]

S1 Table. Baseline demographic and biochemical characteristics of 822 patients in three cohorts of incident dialysis patients, prevalent HD patients and prevalent PD patients.

**Characteristics**	**Incident dialysis (n=523)**	**Prevalent HD (n=212)**	**Prevalent PD (n=87)**
**Age** (years)	55 (33 - 68)	64 (37 - 80)	57 (29 - 80)
**Gender** (male %)	62	58	64
**Residual renal function** (ml/min/1.73 m^2)	6.3 (4.0 - 10.3)	Not determined	4.0 (0 - 8.3)
**Urinary albumin excretion** (mg/24h) [a]	1771 (165 - 6009)	Not available	178 (29 - 1762)
**Cardiovascular Disease** (%)	35	56	21
**Diabetes mellitus** (%)	30	19	20
**Smoking** (%) [b]	51	65	32
**Systolic BP** (mmHg) [c]	148 (122 - 180)	131 (101 - 164)	135 (109 - 172)
**Diastolic BP** (mmHg) [d]	87 (50 - 140)	75 (60 - 92)	80 (67 - 101)
**BMI** (kg/m^2)	24.2 (19.7 - 30.5)	23.9 (18.9 - 30.6)	24.5 (19.1 - 29.1)
**SGA score >1** (%)	32	45	40
**Handgrip strength** (% of control) [e]	83 (51 - 119)	65 (37 - 109)	81 (56 - 108)
**Hemoglobin** (g/L) [f]	105 (87 - 123)	118 (100 - 136)	116 (98 - 131)
**Ferritin** (ng/ml) [g]	275 (88 - 697)	424 (133 - 912)	341 (78 - 850)
**PTH** (ng/L) [h]	232 (60 - 597)	203 (49 - 622)	255 (80 - 554)
**Calcium** (mmol/L) [i]	2.42 (2.07 - 2.77)	2.48 (2.14 - 2.75)	2.26 (2.01 - 2.56)
**Phosphate** (mmol/L) [j]	1.9 (1.3 - 2.6)	1.8 (1.1-2.6)	1.6 (1.2 - 2.2)
**Cholesterol** (mmol/L) [k]	4.8 (3.2 - 7.1)	4.3 (3.0 - 5.8)	4.8 (3.6 - 6.6)
**Triglycerides** (mmol/L) [l]	1.7 (0.9 - 3.3)	1.5 (0.7 - 2.8)	1.4 (0.9 - 2.7)
**Serum Albumin** (g/L)	34 (26 - 40)	35 (29 - 40)	32 (27 - 37)
**Creatinine** (μmol/L)	704 (442 - 1022)	747 (508 - 1013)	681 (474 - 1013)
**hsCRP** (mg/L)	4.0 (0.6 - 28.8)	5.5 (0.5 - 38.7)	1.6 (0.3 - 15.7)
**IL-6** (pg/ml) [m]	5.8 (1.4 - 16.8)	7.8 (1.9 - 23.3)	3.9 (0.8 - 11.0)
**TNF** (pg/ml) [n]	11.5 (7.2 - 21.1)	13.8 (9.1 - 20.7)	14.9 (9.3 - 20.7)
**VCAM-1** (ng/ml) [o]	1322 (836 - 1960)	1679 (1175 - 2058)	Not available
**8-OHdG** (ng/ml) [p]	0.66 (0.25 - 1.10)	1.33 (0.34 - 2.09)	0.44 (0.15 - 0.68)

Abbreviations: MAP, mean arterial pressure; Systolic BP, systolic blood pressure; BMI, body mass index; SGA, subjective global assessment of nutritional status; PTH, parathyroid hormone; hsCRP, high-sensitivity C-reactive protein; IL-6, interleukin 6; PTH, parathyroid hormone; TNF, tumor necrosis factor; VCAM-1, vascular cellular adhesion molecule 1; 8-OHdG, 8-hydroxy-2-deoxyguanosine.

[a] n=325; [b] n=678; [c] n=703; [d] n=703; [e:] n=781; [f] n=821; [g:]n=687; [h] n=725; [i] n=790; [j] n=785; [k] n=817; [l] n=815; [m] n=789; [n] n=754; [o]=480; [p] n=528.
