# Supplementary material for: The higher mortality associated with low serum albumin is dependent on systemic inflammation in end-stage kidney disease
Source: PLoS One. 2018 Jan 3;13(1):e0190410. doi: 10.1371/journal.pone.0190410 (PMC5752034; doi:10.1371/journal.pone.0190410)
Supplement: S2 Table — (PDF) [file pone.0190410.s002.pdf]

S2 Table. All-cause mortality risk associated with low S-Alb <35 g/L and high hsCRP> 3 mg/L (Group 4) during 60 months of follow-up in CKD stage 5, **without PD patients** (n=735) without and with imputed data for smoking status, mean BP and %HGS.

S2a Table. All-cause mortality risk associated with low S-Alb <35 g/L and high hsCRP> 3 mg/L (Group 4) during 60 months of follow-up in CKD stage 5, **without PD patients** (n=735).

	**Adjusted imputed HR (95% CI)**	**p**
**Group 2**<br>Low albumin/ Normal hsCRP	1.19 (0.69 – 2.08)	0.53
**Group 3**<br>Normal albumin/ High hsCRP	1.35 (0.84 – 2.18)	0.21
**Group 4**<br>Low albumin/ High hsCRP	**1.71 (1.10 - 2.66)**	**0.01**

Data are presented as hazard ratios (HR) with 95% confidence interval (CI) adjusted for confounding factors (age, gender, DM, SGA, GFR and renal replacement technique), using Group 1 as reference.

S2b Table. All-cause mortality risk associated with low S-Alb and high hsCRP (Group 4) during 60 months of follow-up in CKD stage 5, **without PD patients** (n=735).

	**Adjusted imputed HR (95% CI)**	**p**
**Group 2**<br>Low albumin/ Normal hsCRP	1.17 (0.67 – 2.02)	0.58
**Group 3**<br>Normal albumin/ High hsCRP	1.29 (0.79 – 2.07)	0.30
**Group 4**<br>Low albumin/ High hsCRP	1.46 (0.94 - 2.29)	0.09

Data are presented as hazard ratios (HR) with 95% confidence interval (CI) crude and adjusted for confounding factors (age, gender, DM, SGA, GFR, mean BP, %HGS and renal replacement technique), using Group 1 as reference. Imputed data were used for smoking status in 128 patients, mean BP in 118 patients and %HGS in 40 patients.
