# Supplementary material for: The higher mortality associated with low serum albumin is dependent on systemic inflammation in end-stage kidney disease
Source: PLoS One. 2018 Jan 3;13(1):e0190410. doi: 10.1371/journal.pone.0190410 (PMC5752034; doi:10.1371/journal.pone.0190410)
Supplement: S3 Table — (PDF) [file pone.0190410.s003.pdf]

S3 Table: All-cause mortality risk associated with low S-Alb and high hsCRP (Group 4) during 60 months of follow-up, adjusting for blood pressure instead of CVD (n=822).

	**Crude HR (95% CI)**	**p**	**Adjusted imputed HR (95% CI)**	**p**
**Group 2** Low albumin/ Normal hsCRP	1.63 (0.98 - 2.73)	0.05	1.04 (0.61 – 1.76)	0.89
**Group 3** Normal albumin/ High hsCRP	2.30 (1.46 - 3.62)	<0.001	1.35 (0.85 – 2.13)	0.21
**Group 4** Low albumin/ High hsCRP	**3.35 (2.22 - 5.04)**	**<0.001**	**1.66 (1.09 - 2.52)**	**0.01**

Data are presented as hazard ratios (HR) with 95% confidence interval (CI) crude and adjusted for confounding factors (age, gender, mean BP, DM, smoking, SGA, GFR and renal replacement technique), using Group 1 as reference. Smoking status was imputed in 144 patients and mean BP in 119 patients.
