# Supplementary material for: The higher mortality associated with low serum albumin is dependent on systemic inflammation in end-stage kidney disease
Source: PLoS One. 2018 Jan 3;13(1):e0190410. doi: 10.1371/journal.pone.0190410 (PMC5752034; doi:10.1371/journal.pone.0190410)
Supplement: S4 Table — (PDF) [file pone.0190410.s004.pdf]

S4 Table. All-cause mortality risk associated with low S-Alb <35 g/L and high hsCRP ≥3 mg/L (Group 4) during 60 months of follow-up (n=822) without and with adjustments for calcium x phosphate product, cholesterol, triglycerides and hemoglobin.

S4a Table. All-cause mortality risk associated with low S-Alb <35 g/L and high hsCRP ≥3 mg/L (Group 4) during 60 months of follow-up (n=822).

	**Adjusted imputed HR (95% CI)**	**p**
**Group 2**<br>Low albumin/ Normal hsCRP	1.34 (0.84 – 2.13)	0.22
**Group 3**<br>Normal albumin/ High hsCRP	1.33 (0.87 – 2.03)	0.18
**Group 4**<br>Low albumin/ High hsCRP	**1.60 (1.08 - 2.36)**	**0.01**

Data are presented as hazard ratios (HR) with 95% confidence interval (CI) crude and adjusted for confounding factors (age, gender, DM, SGA, GFR, renal replacement technique and **%HGS)**, using Group 1 as reference. Imputed data was used for: smoking status in 144 patients, mean BP in 119 patients and %HGS in 41 patients.

S4b Table. All-cause mortality risk associated with low S-Alb <35 g/L and high hsCRP ≥3 mg/L (Group 4) during 60 months of follow-up (n=822).

	**Adjusted imputed HR (95% CI)**	**p**
**Group 2**<br>Low albumin/ Normal hsCRP	1.04 (0.62 – 1.79)	0.86
**Group 3**<br>Normal albumin/ High hsCRP	1.25 (0.79 – 2.01)	0.34
**Group 4**<br>Low albumin/ High hsCRP	1.43 (0.94 - 2.20)	0.11

Data are presented as hazard ratios (HR) with 95% confidence interval (CI) crude and adjusted for confounding factors (age, gender, DM, **smoking**, mean BP, SGA, GFR, renal replacement technique, %HGS, Ca x PO4, cholesterol, triglycerides and hemoglobin), using Group 1 as reference. We have used imputed data for smoking status in 144 patients, mean BP in 119 patients, %HGS in 41 patients, calcium x phosphate product in 36 patients, cholesterol in 5 patients, triglycerides in 7 patients and hemoglobin in one patient.
