# Supplementary material for: The higher mortality associated with low serum albumin is dependent on systemic inflammation in end-stage kidney disease
Source: PLoS One. 2018 Jan 3;13(1):e0190410. doi: 10.1371/journal.pone.0190410 (PMC5752034; doi:10.1371/journal.pone.0190410)
Supplement: S5 Table — (PDF) [file pone.0190410.s005.pdf]

S5 Table. Association of serum albumin as continuous variable with mortality risk for high and low hsCRP (n=822).

	**Adjusted HR (95% CI)**	**p**
Serum albumin g/L in ≥3 mg/L hsCRP	**0.96 (0.93 – 0.99)**	**0.02**
Serum albumin g/L in <3 mg/L hsCRP	0.96 (0.91 – 1.03)	0.26

Data are presented as hazard ratios (HR) with 95% confidence interval (CI) adjusted for confounding factors (age, gender, DM, SGA, GFR and renal replacement technique).
