# Supplementary material for: The higher mortality associated with low serum albumin is dependent on systemic inflammation in end-stage kidney disease
Source: PLoS One. 2018 Jan 3;13(1):e0190410. doi: 10.1371/journal.pone.0190410 (PMC5752034; doi:10.1371/journal.pone.0190410)
Supplement: S6 Table — (DOCX) [file pone.0190410.s006.docx]

**S6 Table.** All-cause mortality risk associated with serum albumin with and without adjustment for hsCRP (n=822).

|  | **Adjusted HR (95% CI)** | **p** |
| --- | --- | --- |
| Serum albumin g/L | **0.96 (0.93 – 0.98)** | **0.003** |
| Serum albumin g/L adjusted for hsCRP mg/L | **0.96 (0.94 – 0.98)** | **0.008** |

Data are presented as hazard ratios (HR) with 95% confidence interval (CI) adjusted for confounding factors (age, gender, DM, SGA, GFR and renal replacement technique).
