# Supplementary figures and images for: The higher mortality associated with low serum albumin is dependent on systemic inflammation in end-stage kidney disease
Source: PLoS One. 2018 Jan 3;13(1):e0190410. doi: 10.1371/journal.pone.0190410 (PMC5752034; doi:10.1371/journal.pone.0190410)

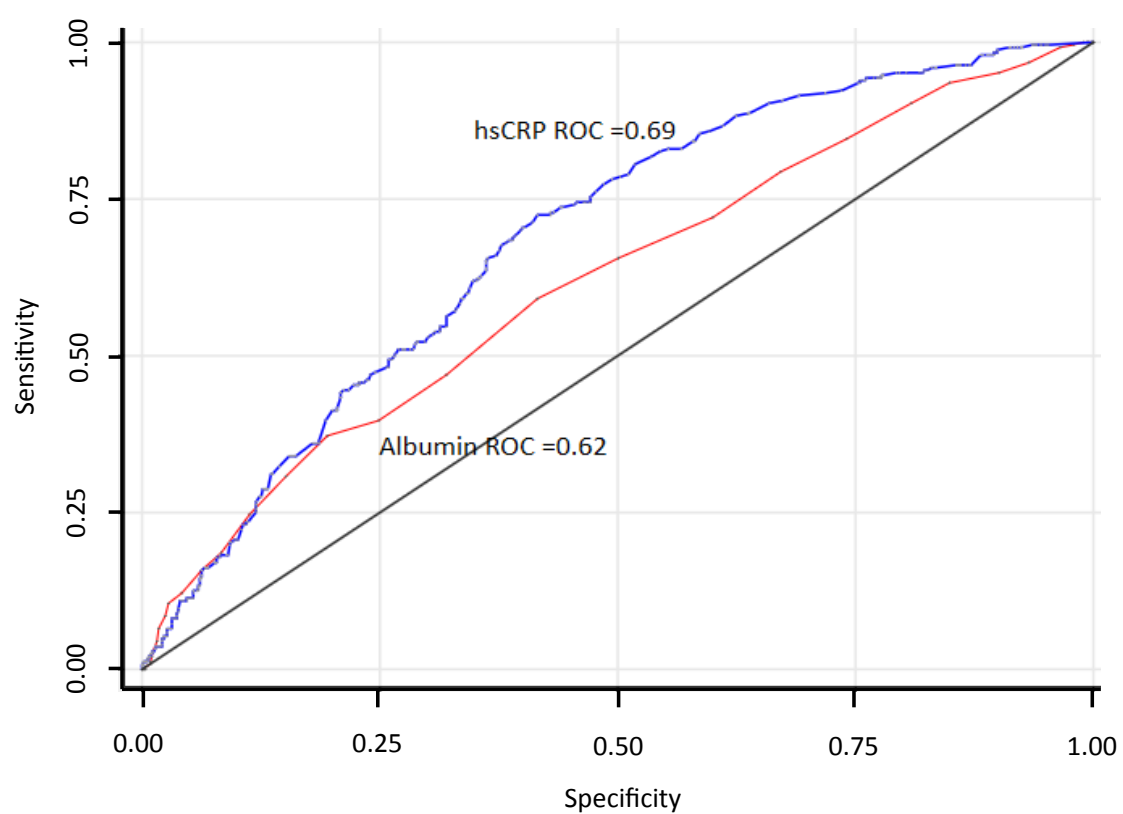

**S1 Fig.** ROC analysis of serum albumin and hsCRP in relation to all-cause mortality.

Supplement: S1 Fig — (PDF) [file pone.0190410.s007.pdf]
